# Insertion methods and gap/void formation in atraumatic restorative technique: A micro-CT analysis

**DOI:** 10.1590/0103-6440202305422

**Published:** 2023-10-27

**Authors:** Paulo Henrique dos Santos Belo, Plinio Mendes Senna, Cesar dos Reis Perez

**Affiliations:** 1 Private Dentist, Rio de Janeiro, RJ, Brazil; 2 Department of Prosthesis, School of Dentistry of State University of Rio de Janeiro, Rio de Janeiro, RJ, Brazil.

**Keywords:** dental caries, dentistry, glass-ionomer cement, wettability, micro-CT

## Abstract

Wetting the dentin is critical to atraumatic restorative treatment. The conventional insertion can be challenging when using high-viscosity glass-ionomer cement. This study evaluated the formation of gaps and voids after three insertion methods using micro-CT. Teeth underwent removal of occlusal and proximal caries through the atraumatic restorative treatment technique. Then, they were fixed in an artificial dental arch to simulate the clinical condition and were restored using three insertion methods: conventional, Centrix injection, and double-filling. Previous dentin conditioning procedures, steel matrix and wooden wedge application, and post-insertion procedures (digital compression and surface protection) were the same. The material was inserted using a manual instrument in the conventional technique and was inserted with a syringe in the Centrix injection group. In the double-filling technique, the tooth received a first layer of a flowable ionomer (through modifying the powder/liquid ratio), and a second layer (with standard ratio) was applied before the final set of the first one. A micro-CT unit scanned each tooth before and after restoration. Each cavity was defined as the volume of interest, and the volumes of gaps and voids were calculated. Data were analyzed using one-way ANOVA and Tukey posthoc test (p < .05). Double-filling had improved filling volume with lower values for gap volume, followed by Centrix injection. The conventional technique had the highest gap volume. No statistically significant difference was observed for void volume. Double-filling demonstrated fewer gaps, followed by Centrix injection, which is critical to obtain better adhesive, remineralizing, and antibacterial activities.

## Resumo

O molhamento da dentina parcialmente desmineralizada no fundo da cavidade dentária é fundamental para o sucesso do tratamento restaurador atraumático. No entanto, o método de inserção convencional pode ser desafiador ao usar cimento de ionômero de vidro de alta viscosidade. Este estudo avaliou a formação de *gaps* e vazios internos após três métodos de inserção de cimento de ionômero de vidro de alta viscosidade usando micro-CT. Dez terceiros molares foram submetidos à remoção de cárie oclusal através da técnica de tratamento restaurador atraumático e complementação proximal (com ou sem lesão pré-existente). Em seguida, foram fixados em uma arcada dentária artificial para simular a condição clínica. Os dentes foram restaurados usando a técnica de tratamento restaurador atraumático com três métodos de inserção: convencional, injeção Centrix e dupla inserção. Os procedimentos anteriores de condicionamento dentinário, inserção de matriz de aço, aplicação de cunha de madeira e procedimentos pós-inserção do material (como compressão digital e proteção de superfície) foram os mesmos para os três grupos testados. O material foi inserido com instrumento manual na técnica convencional, seguindo a metodologia clássica do tratamento restaurador atraumático. O material foi inserido com um dispositivo desenvolvido especificamente para esse fim na técnica de inserção com seringa tipo Centrix. Na técnica de dupla obturação, o dente recebeu uma primeira camada do cimento de ionômero de vidro mais fluido (obtido através da modificação da relação pó/líquido), e uma segunda camada (com relação pó/líquido padrão) foi aplicada antes da presa final da primeira. Um micro-CT escaneou cada dente antes e depois da restauração. Cada cavidade foi definida como o volume de interesse, e os volumes de *gaps* e vazios foram calculados. Os dados foram analisados por ANOVA one-way e teste post-hoc de Tukey com nível de significância de 5%. A dupla inserção melhorou significativamente o volume de preenchimento com valores percentuais mais baixos para o volume do *gap*, seguido pela injeção com seringa tipo Centrix. A técnica convencional apresentou o maior percentual de volume de *gap*. Nenhuma diferença estatisticamente significativa foi observada para o volume de vazios internos. O grupo de dupla inserção demonstrou menor formação de *gaps*, seguido pelo grupo de injeção com seringa tipo Centrix, que é fundamental para obter melhores atividades adesivas, remineralizantes e antibacterianas.

## Introduction

*Minimal intervention dentistry*is a term first coined by Dawson and Makinson ^1^ that represents a philosophy that emphasizes maintaining tooth function for life through treatments that prioritize maximal effectiveness with minimal intervention. Although it can be applied in other areas of oral health, such as periodontology, oral rehabilitation, and oral surgery, this mindset highly influences the management of dental caries ^2^. Atraumatic restorative treatment is directly linked to the minimal intervention dentistry philosophy. This treatment was developed in the mid-1980s to prevent the extraction of decayed teeth in patients in outreach areas where resources such as electricity and rotary dental equipment were not easily accessible ^3^. In addition, people who are elderly, medically compromised, or dentophobic and have challenges accessing dental care can benefit from the atraumatic restorative treatment approach ^4^.

Atraumatic restorative treatment aims to control the biofilm in the tooth instead of removing it from within the cavity, protecting the pulp-dentin complex and arresting the carious lesion by sealing it. The decomposed (previously named “infected”) dentin is removed because it serves no other purpose, while the demineralized (previously named “affected”) dentin is maintained because it can remineralize, as observed in both*in vitro* ^5^ ^,^ ^6^ ^,^ ^8^ ^,^ ^9^and*in vivo studies* ^10^ ^,^ ^11^. Unlike modern adhesive systems, which have a significantly lower bond strength to caries-affected dentin than sound dentin, high-viscosity glass-ionomer cement has a similar bond strength to normal and caries-affected dentin ^12^ ^,^ ^13^ ^,^ ^14^. However, the sealing ability of the restorative material depends on adequate contact with the inner walls of the tooth cavity.

High-viscosity glass-ionomer cement was developed explicitly for atraumatic restorative treatment. It exhibits better mechanical properties than its predecessors do, medium-viscosity glass-ionomer cement, and resin-modified glass-ionomer cement ^15^ ^,^ ^16^ ^,^ ^17^. However, clinical studies have not demonstrated that high-viscosity glass-ionomer cement performs better than other restorative materials ^18^. This contradiction may be due to their brittleness and complex handling requirements ^19^ ^,^ ^20^, which can lead to internal gaps that affect their adhesion and, thus, the restoration’s remineralizing and antimicrobial potential.

It is critical to achieving excellent dentin structure wetting by the high-viscosity glass-ionomer cement, which prevents internal gaps and optimizes adhesion. Likewise, a good insertion technique can contribute to less porosity restoration. 

Analogously, we consider voids as internal spaces in the body of the material, which can weaken the restoration. Thus, this study evaluated the influence of three insertion methods (traditional, with syringe injector, and double-filling, all followed by digital compression) on the formation of gaps and voids in high-viscosity glass-ionomer cement atraumatic restorative treatment restorations through micro-CT**.**

The research's null hypothesis was no difference in the gap or void volume considering the three techniques.

## Materials and methods

The Ethics Committee for Human Research at Pedro Ernesto University Hospital approved this study (CEP HUPE - CAAE: 10522918.2.0000.5259).

An effect size of 0.03 and a standard deviation of 0.04 were estimated based on a pilot study. These values were input into the G*Power software program (version 3.1.9.7; Heinrich-Heine-Universität Düsseldorf, Düsseldorf, Germany) together with an alpha-type error rate of 5% and a beta power of 80%. The software indicated that nine samples per group were sufficient to observe a significant effect. We increased this by 10% to account for possible problems during the experiment. 

Ten third molars extracted for orthodontic reasons and presenting occlusal carious lesions with or without proximal involvement were selected from the Human Teeth Bank of the School of Dentistry at the State University of Rio de Janeiro. Although there is no standard preparation, according to the ATR, an aspect that was also observed was the extent and involvement of the carious lesion. Teeth with very extensive lesions (outside the scope of the technique) were not selected. Specimens were examined for cracks and fractures using a Zeiss Stemi 508 stereo microscope (Oberkochen, Germany). 

**Figure 1:**
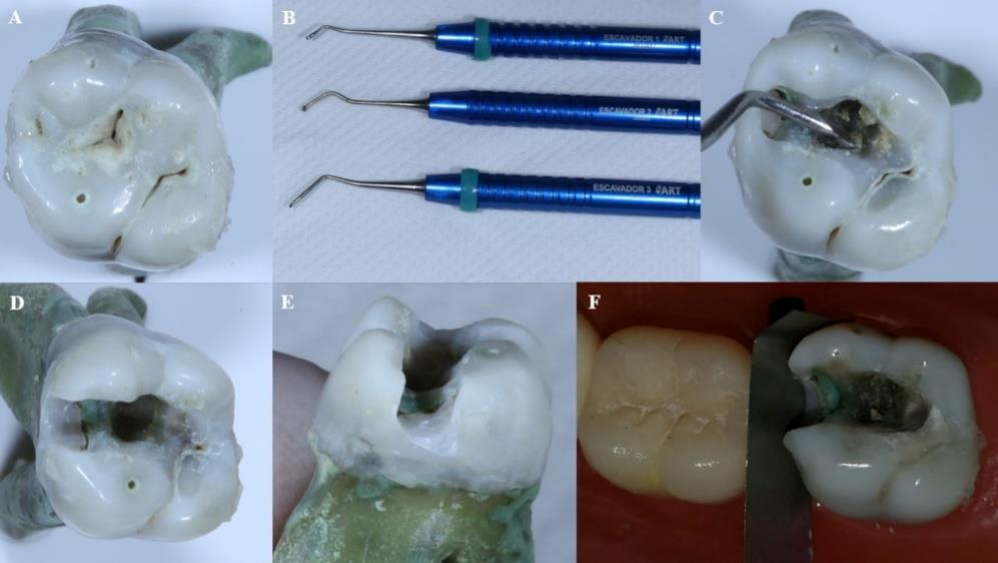
A) Occlusal view of a third molar with an occlusal and mesial carious lesion. B) Set of excavators for ATR used for selective removal of carious tissue. C) Removal of decayed tissue with an excavator for ART. D) Occlusal view of the proximal cavity extension. E) Proximal view of the proximal cavity extension. F) Finished cavity and insertion of steel matrix and wooden wedge.

The three restorative methods evaluated in this study were performed on each tooth. First, the carious tissue was removed following the protocol for atraumatic restorative treatment. Removal of softened tissue started from the center of the lesion. If the lesion was small, a dental hatchet was used to widen the entrance for access, and all the soft demineralized dentine was removed from the enamel-dentin junction (ATR Excavators, Duflex, Petrópolis, RJ, Brazil). After completing carious lesion removal, a proximal extension of the cavity was prepared using a #2114 spherical diamond bur (Shofu, São Paulo, SP, Brazil) following the lingual buccal width of the occlusal box, regardless of whether there is a previous proximal lesion (figure 1). The bur was changed after every five preparations.

**Figure 2:**
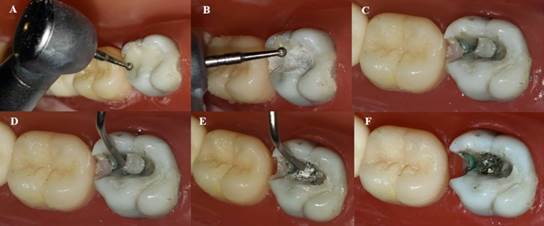
A) Beginning of restoration removal after scanning with a diamond bur 2112; B) careful removal of restorative material with a diamond bur in progress; C) diamond bur removal step completed; D) Beginning of restoration removal with excavators; E) careful removal of restorative material with an excavator in progress; F) Complete restoration removal.

Removal of decayed tissue was only performed before the first randomly selected representative group was created. The same teeth were used for the other groups by carefully removing restorative material. A randomization table was used to determine in which order each restorative technique would be implemented. Before the first restorative procedure, each sample was submitted to micro-CT analysis (Skyscan 1174; Bruker, Kontich, Belgium) to verify the cavity dimensions. After analyzing the restorations of the first group through micro-CT, the restorative material was carefully removed using a diamond bur and hand excavators to allow the execution of a new restorative technique, avoiding any additional dental tissue loss (Figure 2). The same procedure was performed after micro-CT analysis of the second restorative technique group to allow the realization of the third group.

### Restorative procedures and study groups

The restorative procedures were performed individually, each tooth in a dental dummy to simulate clinical conditions. Putty addition silicone (Panasil Putty Soft; Ultradent, South Jordan, UT, USA) was inserted into the dental dummy’s artificial dental socket to allow for stabilization during the procedure and enable reinsertion in the same position during the restorative procedures of the three techniques.

"The cavities were restored by a single, previously trained operator with high-viscosity glass-ionomer cement (Fuji IX, GC Europe, Leuven, BE) using the following initial sequence: 1. A metallic matrix band coated with petroleum lubricating jelly and a wedge (TDV, Santa Catarina, Brazil) were applied; 2. The cavities were conditioned with diluted liquid from the high-viscosity glass-ionomer cement for 10 seconds; 3. The cavities were rinsed with water; 4. The cavities were dried with cotton pellets."

**Figure 3:**
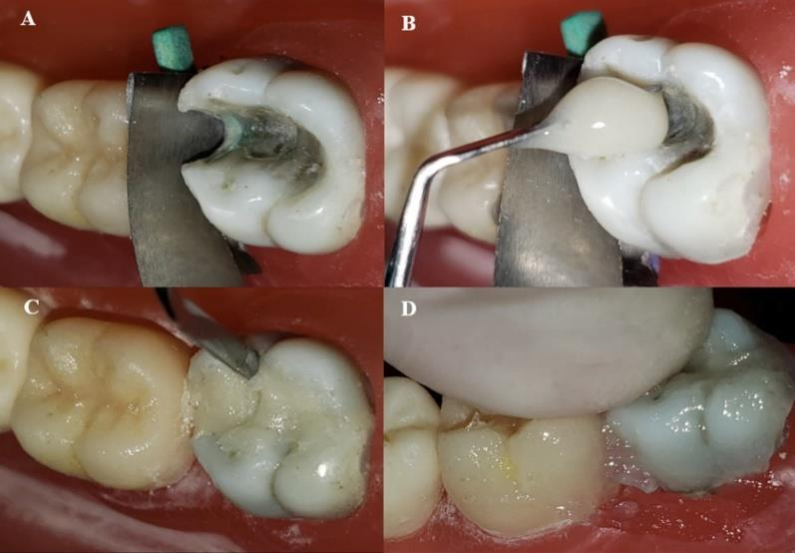
A) Occlusal view of cavity ready for insertion of high viscosity glass-ionomer cement; B) Insertion of restorative material with W1 spatula; C) Removal of metal matrix and wedge and gross excesses; D) Digital compression.

While the high-viscosity glass-ionomer cement employed in the restorations was the same for the three insertion techniques, the protocols for mixing and inserting it differed for each technique. The three insertion methods are described below. Conventional (C) one‐layer technique: Cement was mixed according to the manufacturer’s instructions (powder/liquid ratio: 1 scoop:1 drop). The material was inserted using a manual instrument (W1 Composite Instrument HuFriedy, Chicago, IL, USA). After the loss of brightness on the material’s surface, digital compression was performed for 30 s. The excess material was removed, and a thin layer of petroleum jelly was applied to protect the restoration (Figure 3).

**Figure 4:**
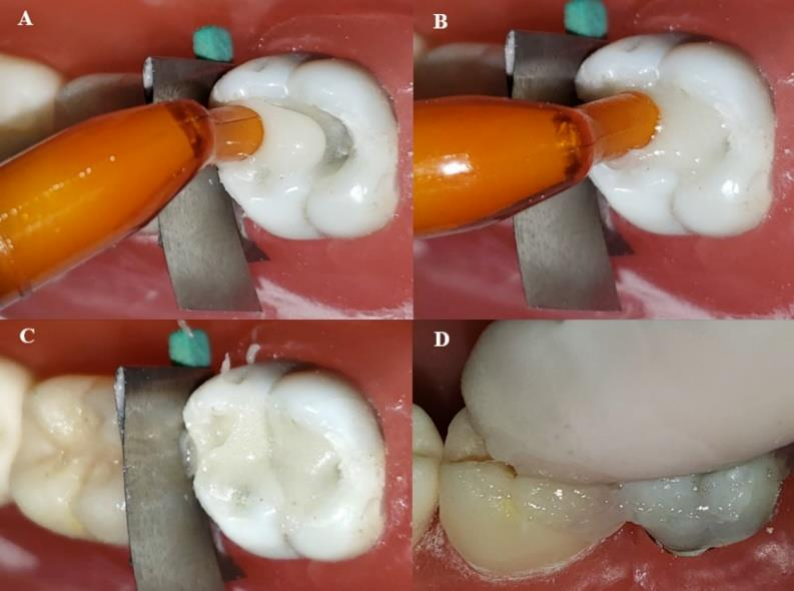
A) Insertion of high viscosity glass ionomer cement with Centrix syringe: start from the proximal box; B) Complete insertion of high viscosity glass ionomer cement with Centrix syringe; C) Removal of gross excess; D) Digital compression after metal matrix and wood wedge removal.

Centrix injection (CI): The traditional insertion method with a spatula was replaced by injection under pressure with AccuDose® High Viscosity Tubes (River Road Shelton, CT, USA). The technique sequence after insertion was the same as that of the conventional technique (Figure 4).

**Figure 5:**
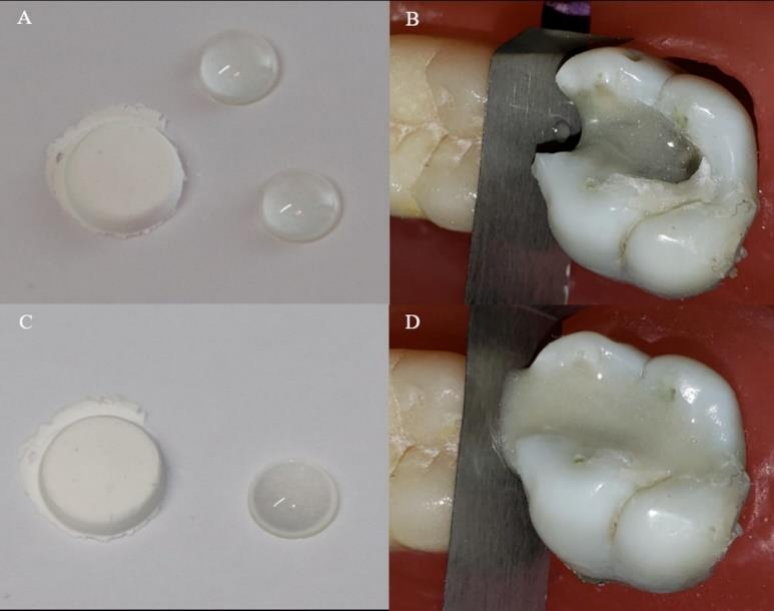
A) Providing a more liquid - -powder/liquid ratio of 1:2 - for insertion of the first layer of high viscosity glass ionomer cement. B) The first flowable layer was applied to the bottom of the entire cavity with a calcium hydroxide placer. C) Usual proportion indicated by the manufacturer. D) The second layer of high-viscosity glass ionomer cement was inserted and compressed digitally.

Double-filling (DF) technique: A first flowable layer (powder/liquid ratio of 1:2) was applied to the bottom of the entire cavity with a calcium hydroxide placer (#8; HuFriedy, Chicago, IL, USA). The second layer was mixed according to the manufacturer’s instructions (powder/liquid ratio: 1:1) and inserted into the cavity before the final set of the first layer using the method employed in the conventional technique (Figure 5).

After the final set, the tooth was removed from the artificial socket and underwent micro-CT analysis. Each tooth had two micro-CT scans for each technique, one before (to check cavity dimension) and one after filling, using a sample holder to allow perpendicular X-ray incidence and a similar tridimensional position during both scans. The operating condition of the micro-CT equipment was 50 kV voltage, 800 μA current, 0.5 mm aluminum filter, 10000 ms exposure time, 16 μm pixel size, and 1304×1024 pixels sensor resolution. Image acquisition was performed using a rotation step of 0.7 degrees and an average of three frames. The scan duration was approximately 120 min. The X-ray projections were reconstructed (NRecon®, version 1.6.9.18; Bruker, Kontich, Belgium) using the following settings: smoothing, 2 (20%); automatic misalignment compensation; ring artifact reduction, 4 (20%); and beam-hardening correction, 40%. Extra care was taken to define the same dynamic range on each tooth’s two scans.

**Figure 6:**
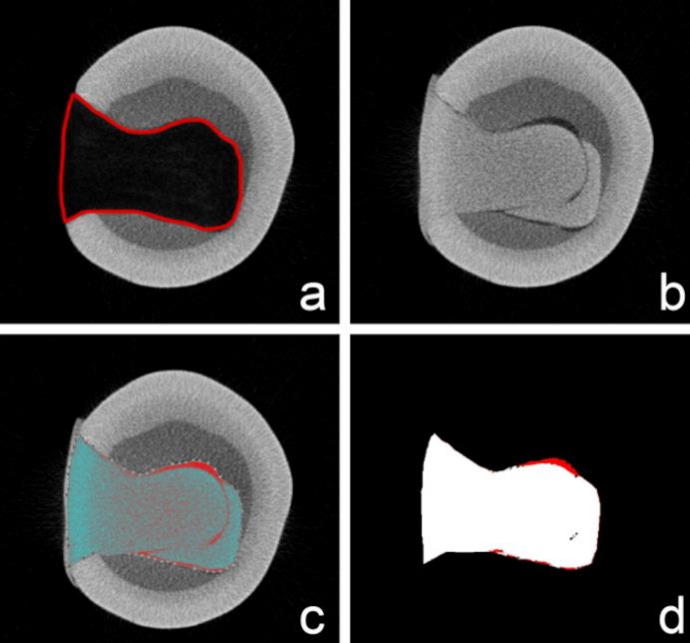
The unrestored tooth was scanned to determine de cavity as the volume of interest (VOI) (a). After restoring, the tooth was scanned again, and the tridimensional registration was performed to fit the previous scanning (b). The VOI was loaded in the restored tooth dataset (c), and after binarization of the image, the volume of gaps and voids was calculated (d).

Each tooth image dataset (before and after filling) was loaded into the DataViewer software program (version 1.5.1.2; Bruker, Kontich, Belgium) for tridimensional registration of the before and after datasets. The volume of interest of the cavity was defined before filling because of similar radiolucency between filler and enamel and applied on the second dataset in the CTAn software program (version 1.14.4.1; Bruker, Kontich, Belgium). The filling material was binarized from the total cavity volume, and the mathematical calculation of the restoration volume, the volume of interface gaps, and voids were then performed using the “3D analysis” tool in the software program. Figure 6 shows the steps in the evaluation methodology.

After verifying the normality of the data with the Shapiro-Wilk test, the dependent variables (restoration, gap, and void volume) were transformed (log10) to reach the parameters of normality. The percentage results of the three techniques’ restoration, void, and gap volumes were compared using a one-way analysis of variance with Tukey’s test (SPSS v.20) for repeated measures. A significance level of 5% was employed.

## Results

The percentage results (mean and standard deviation) are shown in Table 1.

**Table 1:** Percentage results (mean and standard deviation) of the techniques’ restoration, void, and gap volumes

Techniques	Rest		Void		Gap	
	Mean	SD	Mean	SD	Mean	SD
C	97.93	0.89	0.37	0.24	1.70	0.82
CI	98.84	0.70	0.28	0.27	0.88	0.59
DF	99.37	0.21	0.36	0.15	0.27	0.13

Techniques’ abbreviations: C = conventional; CI = Centrix injection, DF = double-filling

**Figure 7:**
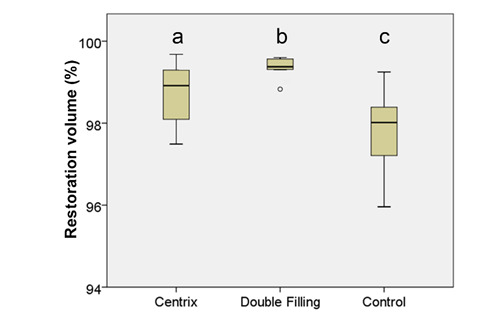
Box plot with medians and standard deviations of the three restorations volume (%) techniques. The different letters indicate statistically significant differences (*p*<0.05) between the techniques.

**Figure 8:**
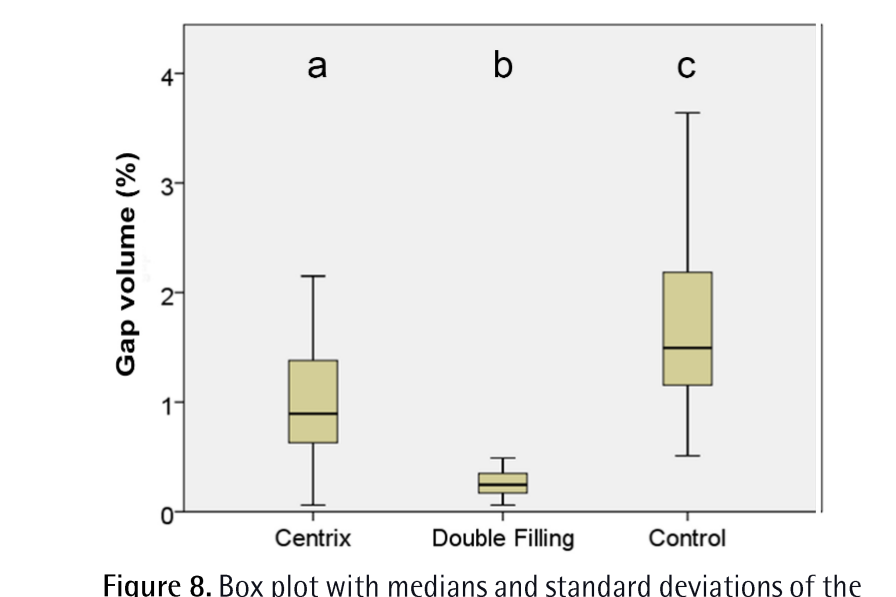
Box plot with medians and standard deviations of the three techniques' gap volume (%). The different letters indicate statistically significant differences (*p*<0.05) between the techniques.

**Figure 9:**
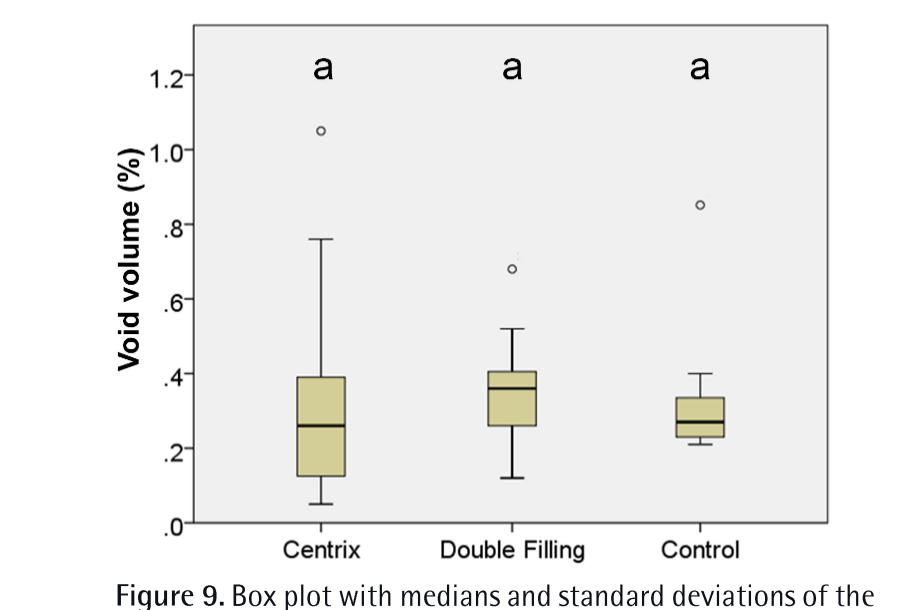
Box plot with medians and standard deviations of the three techniques' void volume (%). Equal letters indicate no significant differences (*p*>0.05) between the techniques.

Figures 7, 8, and 9 present box plots demonstrating the percentage results from the distribution of the restoration techniques: cavity, void, and gap volumes, respectively. The double-filling technique showed the highest (*p*<0.05) restoration volume, followed by the Centrix injection and the conventional techniques (Figure 7). The double-filling technique had significantly (*p*<0.05) lower percentage values for gap volume than the Centrix injection and the conventional techniques, as well as less dispersion of the results, indicating better reproducibility (Figure 8). No statistically significant (*p*>0.05) difference was found between the techniques in the percentage values for void volume (Figure 9).

**Figure 10:**
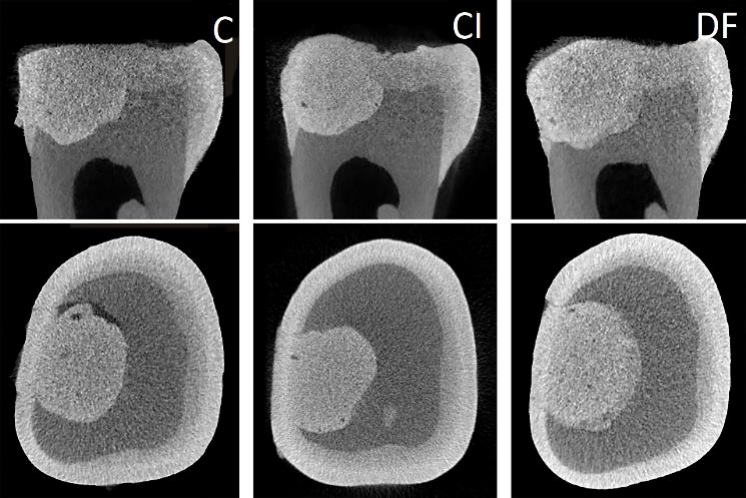
Representative two-dimensional micro-CT** **images of all three techniques from the same tooth: sagittal (upper) and axial (lower) views. The conventional (C) technique shows more evident gap formation. The two layers of material are visible in the double-filling (DF) technique.

**Figure 11:**
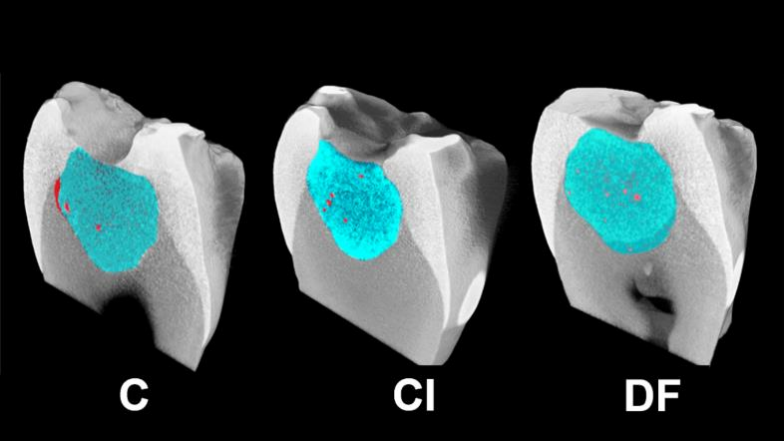
3D models showing gaps and voids (in red) respectively in groups of different insertion techniques: conventional (C), Centrix injection (CI), and double-filling (DF).

Figure 4 displays micro-CT images generated for specimens of the three techniques (conventional, Centrix injection, and double-filling) performed on the same tooth. Both sagittal (upper) and axial (lower) views allow the observation of gaps in the conventional technique. It is also possible to observe the presence of a more fluid layer (less radiopaque) at the bottom of the cavity in the double-filling technique (Figure 10). Figure 11 shows 3D models of the three different insertion techniques: glass ionomer cement is represented by cyan, and gaps and voids are represented by red.

## Discussion

The first null hypothesis that there would be no differences in gap volume between the techniques was rejected, as the double-filling technique demonstrated the lowest gap volume percentage, followed by the Centrix injection and conventional techniques. This result is the primary finding of this work.

Developed to enhance the adaptation of the high-viscosity glass-ionomer cement in a posterior proximal cavity, particularly at the cervical margin ^19^ ^,^ ^20^, the double-filling technique (also known as the two-layer or bi-layer technique) continues to generate controversy regarding its effectiveness. Despite its presentation as a technique that provides relevant solutions and good*in vitro*results ^21^, clinical results must correspond to expectations ^22^ ^,^ ^23^. Close contact with the surface, aided by the hydrophilic nature of the cement and the dentin surface, is essential when considering adhesion. The adhesion that occurs during the wetting stage has been suggested as being caused by the formation of hydrogen bonds between the free carboxylate groups of the cement and the layer of tightly bound water on the surface of the mineral phase of the tooth. These hydrogen bonds are gradually replaced by genuine ionic bonds formed between calcium ions in the tooth and carboxylate groups in the polymer within the cement ^20^. In addition, a micromechanical interlocking occurs due to the formation of short cement tags within the dentin surface and a thin hybrid layer between hydroxyapatite-coated collagen fibrils on the tooth surface and the surface of the freshly placed glass-ionomer cement ^13^. These findings suggest that glass ionomer cement may be self-etching, an effect that arises from its polyalkenoic acid component ^4^ ^,^ ^11^ ^,^ ^12^ ^,^ ^13^ ^,^ ^14^.

Whether any bonding to collagen within the tooth structure occurs during the adhesion of glass ionomer cement has yet to be fully answered. The fact that bonds are stronger to enamel than dentin suggests that any bonds to the organic phase of the tooth are unimportant and that collagen has no role at all. However, as collagen contains amino and carboxylic acid groups, there may be an interaction with the glass ionomer cement’s carboxylate groups, causing some adhesion due to bonding to collagen. However, the evidence suggests that bonds of this type are not particularly important in the adhesion mechanism of glass ionomer cement ^13^. Furthermore, high-viscosity glass-ionomer cement has similar bond strengths to normal and caries-affected dentin ^12^ ^,^ ^13^ ^,^ ^14^.

Studies analyzing the remineralization potential of glass ionomer cement*in vitro* ^5^ ^,^ ^6^ ^,^ ^7^ and*in vivo* ^10^ ^,^ ^11^ have demonstrated that calcium, phosphate, and strontium ion exchanges occur with the glass ionomer cement in deep dentinal lesions following incomplete carious lesion removal. However, there are two essential requirements for this to happen. First, the restoration must provide a total seal to the external environment. Second, intimate contact must be between the glass ionomer cement and the partially demineralized dentin ^13^. It is, however, relevant to emphasize that there is no consensus on this topic, and other studies have found that the lining material is not fundamental to caries arrestment ^16^.

Given the antimicrobial effect of glass ionomer cement on contaminated dentin, the intrinsic characteristics of atraumatic restorative treatment make using glass ionomer cement even more relevant. Cavity size, dentin color, and consistency are not absolute indicators of the microbiological bioburden within an atraumatic restorative treatment cavity. Despite removing the infected layer, viable bacteria have been consistently found in the remaining affected dentin. Consequently, using a material with bacteriostatic properties is indicated, and liners that provide some antibacterial effect to promote an immediate inactivation of microorganisms are beneficial ^24^.

Considering the available evidence and the consistency of high-viscosity glass-ionomer cement, using an initial layer of material with a more fluid consistency and greater flow has advantages, eliciting better wetting to the dentin and a lower occurrence of interfacial gaps. This study confirmed these benefits, as the double-filling technique demonstrated significantly superior results compared to Centrix injection and conventional techniques considering gap volume, as shown in Figure 8. Micro-CT analysis allowed a better comprehension of what occurs internally in the restoration, giving a non-destructive and more real possibility for evaluation. 

Due to the consistency of high-viscosity glass-ionomer cement, the Centrix injection technique was compared to the conventional technique and performed better. However, the extent to which this impacts restoration prognosis remains unknown. Every effort must be made to obtain the best wetting for the dentin walls to optimize the restoration results.

It is important to note that the digital compression method was used in all techniques. A significant advantage of using high-viscosity glass-ionomer cement over composite resin is that it allows the practitioner to use the press-finger technique to place the material into the cavity, leading to a sealant restoration ^25^.

The second null hypothesis confirmed that there would be no difference in void volume between the three techniques. There was some discussion amongst the authors regarding the potential effects of the techniques on the formation of voids. The first point of discussion centered on whether the conventional technique would demonstrate more internal voids, as inserting the material correctly with this technique can be difficult due to the material’s consistency. Better results are expected from the Centrix injection technique. However, this was not observed in our study, as no statistically significant differences between the three techniques were found in void formation. Factors such as the operator’s dexterity and the press-finger technique may have influenced these results ^25^. A second point of discussion related to a possible increase in void volume when using the double-filling technique. Similarly, no differences were observed, potentially due to the same influencing factors.

As a complementary measurement, restoration volume was calculated as the total volume minus the volume of gaps and voids. There was a statistically significant difference in restoration volume between the three techniques.

This study tried to reproduce*in vitro*all the technical steps of the clinical situation. Moreover, it used the same cavities for the three techniques tested, reducing the variability of the study. However, it is essential to highlight that a new clinical study testing the same techniques should be concluded soon, using primary teeth restored and analyzed after exfoliation.

This work has limitations related to*in vitro*simulation and extrapolation of results to clinical situations involving different operators. Despite following a methodology, that simulates clinical conditions during the restorative procedure and adopts sample calculation, the procedure is closely linked to the operator's dexterity and knowledge. In this work, only a professional trained in ART performed all the restorative steps. As glass ionomer cement is a relatively sticky material, even in its high-viscosity version, its use requires a learning curve, and the results may vary significantly with different operators.

Within the limitations of this*in vitro*study, it is possible to conclude that there was a statistically significant difference considering gap formation, rejecting the first null hypothesis: the double-filling technique (also known as the two-layer technique) demonstrated the lower percentage of gap volume, followed by the Centrix insertion technique. The conventional technique showed the highest gap volume. No statistically significant difference in void volume was observed.
